# Application and Communication Optimization Technology of Unmanned Distribution Car under Deep Learning in Logistics Express of COVID-19

**DOI:** 10.1155/2022/5386737

**Published:** 2022-09-17

**Authors:** Xinyue Song, Fengkai Luan

**Affiliations:** ^1^School of Logistics, Yunnan University of Finance and Economics, Kunming 650000, China; ^2^School of Information Engineering, Wuhan University of Technology, Wuhan 430000, China

## Abstract

This work aims to solve the problem that the daily necessities of urban residents cannot be delivered during coronavirus disease 2019 (COVID-19), thereby reducing the possibility of the delivery personnel contracting COVID-19 due to the need to transport medicines to the hospital during the epidemic. Firstly, this work studies the application and communication optimization technology of unmanned delivery cars based on deep learning (DL) under COVID-19. Secondly, a route planning method for unmanned delivery cars based on the DL method is proposed under the influence of factors such as maximum flight time, load, and road conditions. This work analyzes and introduces unmanned delivery cars from four aspects combined with the actual operation of unmanned delivery cars and related literature: the characteristics, delivery mode, economy, and limitations of unmanned delivery cars. The unmanned delivery car is in the promotion stage. A basic AVRPTW model is established that minimizes the total delivery cost without considering the charging behavior under the restriction of some routes, delivery time, load, and other factors. The path optimization problem of unmanned delivery cars in various situations is considered. A multiobjective optimization model of the unmanned delivery car in the charging/swap mode is established with the goal of minimizing the total delivery cost and maximizing customer satisfaction under the premise of meeting the car driving requirements. An improved genetic algorithm is designed to solve the established model. Finally, the model is tested, and its results are analyzed. The effectiveness of this route planning method is proved through case analysis. Customer satisfaction, delivery time, cost input, and other aspects have been greatly improved through the improvement and optimization of the unmanned delivery car line, which has been well applied in practice. In addition, unmanned delivery cars are affected by many factors such as load, and the service time required for delivery is longer. Therefore, this work chooses an unmanned distribution car with strong endurance to improve distribution efficiency. The new hospital contactless distribution mode discussed here will play an important role in promoting future development.

## 1. Introduction

Before the outbreak of coronavirus disease 2019 (COVID-19), people do not fully understand the concept of “no contact.” Most of the current express services are manually transported by couriers, which is laborious and time-consuming, and the labor cost is high. After the outbreak of the epidemic, the implementation of epidemic prevention and control work leads to the inability of express delivery to be delivered in time, and owners are at risk of cross-infection. “Contactless distribution” is a distribution service model launched by enterprises during the fight against the new coronavirus pneumonia epidemic according to the actual operation and internal standards and specifications of the enterprise [1]. Therefore, the implementation of “contactless distribution” has become an important topic for major property enterprises. With the continuous improvement of the level of mechanical scientific research, the application of unmanned distribution cars has gradually developed into the service industry. Artificial intelligence (AI) is gradually infiltrating the field of logistics, completely subverting the traditional logistics model, and becoming a future development trend. In addition, China has also made great progress in “5th Generation Mobile Communication Technology (5G)” network research. 5G base stations are gradually gaining popularity around the world [2]. The integration of “5G” and “AI” will inevitably provide favorable technical conditions for the development of the “contactless” model. The “smart logistics” proposed in the “two sessions” also provide favorable external conditions for the development of “contactless.” The “contactless distribution” is a new type of logistics distribution, which is still in the exploratory stage. The relevant knowledge reserve is not perfect, and further discussion and research are needed [3].

American courier enterprises Instacart and Postmates have introduced the concept of “contactless delivery.” Instacart, a San Francisco-based enterprise, has started a small “entry point” delivery business. Many people like this service, so the platform decides to expand this service to North America [4]. Currently, most of the research on unmanned car delivery focuses on three aspects. On the one hand, some scholars have researched the feasibility of unmanned car distribution, mainly focusing on the impact of unmanned cars on traffic flow and the taxi industry. Alireza and Mahmassanin studied the influence of different types of cars on traffic flow stability through different models. The results showed that unmanned delivery cars positively impacted traffic flow stability [5]. Calvert et al. pointed out that low-level driverless cars slightly negatively impacted traffic flow and road capacity. However, it would positively impact when the proportion of driverless cars exceeded 70% [6]. Hartwell et al. mainly studied the unmanned delivery vehicle in food distribution, compared with the trolley, and analyzed the advantages and disadvantages of the unmanned delivery vehicle [7]. Zhang et al. studied the application of unmanned delivery vehicles in drug delivery, and specifically introduced the role and significance of unmanned delivery vehicles in the process of intelligent drug delivery [8]. Sorigueralll et al. and Chehri and Mouftah believed that autonomous cars could increase road safety, improve traffic efficiency, and reduce emissions of pollutants such as CO_2_ [9, 10]. On the other hand, the research focuses on the car path planning of unmanned cars. Some scholars are interested in the path optimization of unmanned cars. Zhang et al. studied the path optimization problem of unmanned cars by constructing an AVRPTW model under the background that some road sections were prohibited [11]. Yu first proposed the concept of the unmanned car logistics system, focusing on the path planning of unmanned cars considering charging. It was concluded that the unmanned car logistics system was stable through the simulation method [12]. Yan proposed the routing problem of unmanned cars considering driver satisfaction [13]. Moncefllies et al. proposed a path optimization and driving speed optimization problem for driverless trucks in uncertain traffic environments. A two-stage stochastic programming model was established based on the two resource strategies [14]. However, the current research on “contactless delivery” is incomplete. The reform of the “contactless distribution” model is imperative. Therefore, this work studies the application of unmanned delivery cars based on DL in logistics communication in epidemics, hoping to provide a basis for subsequent research in this area.

The innovations are as follows: (1) the route optimization problem of unmanned delivery cars is studied, and the corresponding model and the optimization scheme of the model are given. (2) Quantitative research on the various characteristics of the unmanned distribution car flow reduces the cost of logistics and improves customer satisfaction. (3) The application of unmanned distribution cars in logistics and express delivery under the epidemic situation is studied, which lays a rich theoretical basis for subsequent research in this area.

## 2. Materials and Methods

### 2.1. Research on the Purpose of Unmanned Delivery Cars

As a new vocabulary and new trend in emergency logistics, the term “contactless delivery” has emerged, which will affect the development trend of related industries such as smart express cabinets and unmanned distribution cars. It is widely used in takeaway, fresh food distribution, instant distribution, and hospital distribution of medicines. It is an emerging industry. “Contactless delivery” is an express delivery service at the end of logistics. Before the Wuhan epidemic attracted attention, “contactless delivery” had been widely practiced due to factors such as “last mile” delivery, recipient privacy protection, and user safety. It completes the delivery of goods at the end of express logistics with the help of third-party platforms represented by smart express cabinets, express stations, and collection points. In this epidemic, seeking new ways to prevent infection of medical staff has become an urgent problem to be solved in addition to consolidating traditional infection training and safeguarding protective materials in hospitals. Therefore, the contactless distribution of medicines has been implemented in the special environment of the hospital. Refined and intelligent pharmacy services based on unmanned car drug distribution have been carried out combined with noncontact logistics technology to fully meet the urgent medication needs of patients and minimize the infection risk of medical staff. There are still some difficulties in the technical implementation of “contactless drug delivery due to the particularity of the hospital environment and the relationship between doctors and patients.” The mobile cabin hospital is one example.

As an innovative model of terminal logistics services, “contactless delivery” has changed the routine face-to-face delivery of goods by unmanned delivery cars, meeting the needs of users for the safety of logistics services during this specific period of the epidemic. Similar models have existed before. However, third-party platforms such as smart express cabinets, express stations, and collection points are used to protect the privacy and security of recipients, improve delivery efficiency, and save manpower. The epidemic has made “contactless delivery” popular, and it has also been applied to hospitals. The mobile cabin hospital of the Optics Valley Science and Technology Convention and Exhibition Center is a smart mobile cabin hospital integrating 5G cloud technology. During the epidemic, contactless distribution saves protective materials and enables medical staff to achieve “zero” infection, which is worthy of promotion and reference. It is also suitable for drug dispensing, deployment, and distribution in emergencies, providing a new model for hospital drug distribution services on the front line of the fight against the epidemic.

### 2.2. Unmanned Delivery Car

Unmanned delivery cars, also known as joint cars, can drive autonomously, sense road conditions, interact with users, and provide express or other things to users at any time [15]. Unmanned distribution is a new type of product in the era of intelligent logistics. Its research goal is to solve the problems existing in the current human resource transportation [16]. In the process of logistics and distribution, unmanned distribution cars load and deliver goods to designated locations to achieve distribution. The unmanned cars that are used in logistics and distribution are usually small- and medium-sized unmanned cars compared with ordinary cars. The landing of some unmanned delivery car products in China is summarized in Table 1.

According to the data analysis of the current unmanned delivery cars on the market, the current unmanned delivery cars in the market have five main characteristics: various sizes, low-speed driving, mainly charging, multiple endurance, and multiple types of loading [17]. The parameters of several major unmanned transport cars are listed in Table 2.

**Figure 1:**
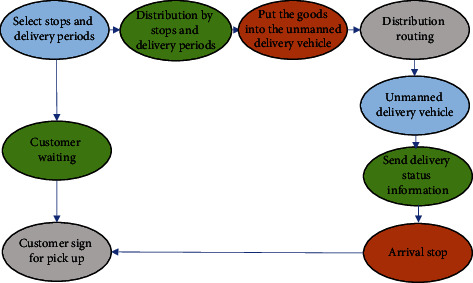
Schematic diagram of the delivery mode of unmanned delivery cars.

The delivery operation mode of unmanned delivery cars can be simply described as follows. Customers choose a site and time during the delivery process. The delivery personnel sort the required goods at the delivery site and place them on the shelves of the automatic delivery car [18]. The driverless car automatically arrives at the designated location and sends a message to the recipient containing the arrival time and location before arriving. When the mail arrives, the driverless car sends a text message again that includes a pickup code. After the sending code is entered at the receiving end, it will be automatically sent to the next end [19]. The overall model of transportation is shown in Figure 1.

**Figure 2:**
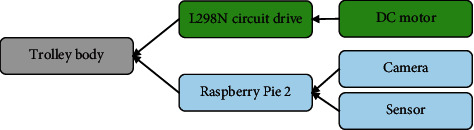
Structure diagram of the components of the autonomous driving smart car.

The system is mainly composed of four modules: power module, control module, drive L298m module, and information acquisition module.  Figure 2 suggests the structure diagram of the components of the autonomous driving smart car.

The core part of the control adopts Raspberry Pi. In this design, Raspberry Pi 2 is used as the core control component. The Raspberry Pi board is connected to the USB camera to obtain real-time picture information. Raspberry Pi is connected to the L298N double H-bridge circuit driver chip. The forward, backward, and steering of the car are mastered by controlling the forward and reverse rotation of the motor [20]. Raspberry Pi is mainly connected to the hardware through the GPIO port (input/output port) to exchange data, control the work of the hardware, and read the working status signal of the hardware.

The data collection of unmanned delivery cars mainly relies on USB cameras. The transfer rate of USB is faster than that of serial and parallel ports. Moreover, a signal processor can be installed in it as a cache, and the power consumption is also very low [21]. The camera is directly connected to the USB port of Raspberry Pi. Raspberry Pi is used to start and control cameras and capture images for analysis. The transmission module of the car consists of an electric motor and a transmission disc motor. The drive disc is driven by L298N double H-bridge direct-current motors. The front and rear movement of the smart car can be realized through the rotation of the two wheels. Steering can also be accomplished by the rotation of a single wheel [22].

The system structure design of the intelligent car is mainly carried out by the car for data collection. The PC uses DL algorithms for data processing and provides correct direction indications as needed [23]. The PC is the berry pie of the control panel. A Socket server is installed on the car to collect data from the camera and give commands to the controller and control terminal. Meanwhile, using Motion as a monitoring system on the PC side can observe the images of the surveillance cameras in real-time. Then, the image is analyzed using DL methods to identify obstacles. The distance between the car and the obstacle is obtained using the ultrasonic sensor. The best directions are provided based on the information. In addition, the transmission system of the car also needs PC commands to operate [24].

### 2.3. A Basic Model for Route Planning of Unmanned Delivery Cars

#### 2.3.1. Parameter Introduction

  0 and *N* +  1: distribution center. 0 represents the start of delivery, and *N* + 1 represents the end of delivery.
 
*C*: customer point collection
  Co: the set of customer points and delivery starting points, *Co* = *CU*{0}
 
*C *_*N*+1_: the set of customer points and delivery termination points, *C*_*N*+1_ = *CU*{*N* + 1}
 
*C *_*d*_: interrupt client point set
 
*K*: a collection of unmanned delivery cars, *K* = {1, 2,…, *k*}
 
*d*_*ij*_^*k*^: the distance between the unmanned delivery car *k* from the node iEN to the node *j*∈*N*, *i*≠*j*
 
*Q*_*i*_^*k*^: the total weight of the cargo carried by the unmanned delivery car *k* at node *i*
 
*M*: the number of unmanned delivery cars held by the delivery station
 
*q *_*i*_: the order weight of customer point i
  [Ei, Li]: the acceptable delivery time window for customer *i*
  [*e*_*i*_, *l*_*i*_]: delivery time window scheduled by customer *i*
 
*α*: penalty coefficient for earlier than the predetermined time
 
*β*: penalty coefficient for later than the predetermined time
 
*Q *_max_: the maximum carrying capacity of the unmanned delivery car
 
*V*: the driving speed of the unmanned delivery car. It is a constant.
 
*T *_*ij*_: the time taken by the unmanned delivery car from node *i* to node *j*, *T*_*ij*_ = *d*_*ij*_/*V*
 
*S *_*i*_: the time when the unmanned delivery car arrives at node *i*
 
*S *_*ti*_: the service time of the unmanned delivery car at node *i*
 
*μ *_*i*_: the waiting time of the unmanned delivery car at customer point *i*
 
*t *_*i*_: the time for the unmanned delivery car to arrive at customer point *i*
 
*Z*: the total delivery cost
 
*C *_1_: the fixed cost of the delivery car
 
*C *_2_: transportation cost per unit distance
 
*P*(*t*_*i*_): penalty cost incurred at customer point *i*
 
*x*_*ij*_^*k*^: it is one when the unmanned delivery car *k* directly goes from node *i* to node *j*. Otherwise, it is zero.

The waiting time of the car is the time that the unmanned delivery trolley waits for the material to be shipped and the time that the customer claims the material in the process of transporting the material.

The service time of a car is the entire time period from when the car is equipped with the substance and starts to depart until the car completes the transport of the substance.

#### 2.3.2. The First Is the Car Route Planning Problem

In the car routing problem, the most basic problem is the car routing problem considering load constraints [25]. The problem can be described as follows. A distribution center has *K* cars that serve *N* in-demand customers. Then, the distribution center must design the transportation planning and route for each car to meet the requirements of all customers and ensure that the number of cars allocated does not exceed the capacity limit of the car [26]. The following basic assumptions must be met to make the problem simple:

1. Each car starts from the distribution center and finally returns to the distribution center
2. There is only one chance per autonomous car
3. All cars must not exceed the maximum carrying capacity

From a graph theory perspective, the CVRP can be described as follows. There is a set of directed graphs *G*=(V, *E*). *V*={0,1,2,…, *N*} refers to the set of all points. *E* = {*G*, *j*: *i*,: *J* ∈ V, *i*≠*j*} refers to the set of delivery routes between two adjacent customers. The related parameter settings are as follows:

*V*: customer set, *V* = {1, 2,…, *n*}
*V*′: the set of customers and distribution centers, *V*′ = *VU*{0}
*K*: the number of cars owned by the distribution center
*Q*: maximum car capacity
*qi*: the demand of customer *i*
*dij*: the distance between nodes *i* and *j*
*gij*: the weight of the car on the arc (*i* : *j*)
  (1) $$\begin{array}{c} x_{ij}=\left\{\begin{array}{c} 1,car\;passes\;arc\left(i,j\right)\\ 0,car\;do\;es\;not\;pass\;arc\left(i,j\right)\text{.} \end{array}\right. \end{array}$$

Here, when the unmanned delivery car directly goes from node *i* to node *j*, one is recorded. Otherwise, zero is recorded. Therefore, *x*_*ij*_ ∈ [0,1].

The model is

(2) $$\begin{array}{c} \min\underset{ieV}{\sum }\underset{ieV}{\sum }x_{ij}d_{ij}\text{,} \end{array}$$

subject to

(3) $$\begin{array}{c} \begin{array}{c} \underset{jeV,i\neq j}{\sum }x_{ij}=1,\forall i\in V\text{,}\\ \underset{jeV,i\neq j}{\sum }x_{ij}=\underset{jeV,i\neq j}{\sum }x_{ji},\forall i\in V\text{,}\\ \underset{jeV,i\neq j}{\sum }x_{oj}\leq K\text{,}\\ \underset{jeV,i\neq j}{\sum }{g_{ji}-g}_{ij}=q_{i},\forall i\in V\text{,}\\ 0\leq g_{ij}\leq Q\times x_{ij}\forall i\in V,j\in V,i\neq j\text{,}\\ x_{ij}\in \left\{0\text{,}\right.\left.1\right\}\forall i\in V,j\in V,i\neq j\text{.} \end{array} \end{array}$$

1. The smallest total distance is represented
2. Each customer has only one visit
3. The access and balance of cars are limited
4. The car usage is restricted less than or equal to the car it owns
5. A change in car load is represented
6. At any node, the load of the car shall not exceed the maximum load of the car
7. The determinants are identified

#### 2.3.3. The Second Is the Car Route Planning Problem considering the Time Window

The CVRP is introduced into the time window, and the original problem becomes VRPTW. The “time window” means the delivery date agreed upon by the customer and the distribution center. The time window of client point *i* can be represented by [*ET*_*i*_,*LT*_*i*_]. In this time window, *ET* and *LT* are the earliest and latest times, respectively, so the delivery car can reach the customer *i*. Logistics services continue to develop, and customers can determine the delivery time by preordering. The traditional car routing problem is the time window-based car routing problem [27]. Time windows can be divided into “hard time windows,” “soft time windows,” and “mixed time windows” according to the strictness of time windows [28]. Unlike the CVRP, the VRPTW model requires adding a time limit to the base model of CVRP. The relevant parameters are as follows:

 
*m*: transportation cost per unit distance
 
*v*: car speed
  1: the time when the car arrives at *i*
 
*w*_*i*_: the waiting time of the car at *i*
 
*w*_*j*_: the service time of the car at *i*
  [*ET*_*i*_, *LT*_*i*_]: time window at node *i*
 
*Pi*: penalty cost at customer *i*

The base model is

(4) $$\begin{array}{c} \min\;=\underset{ieV}{\sum }\underset{ieV}{\sum }{mx}_{ij}+\underset{ieV}{\sum }p\left(t_{i}\right)\text{.} \end{array}$$

The added constraints are

(5) $$\begin{array}{c} \begin{array}{c} ET_{I}\leq t_{i}\leq {LT}_{i}\forall i\in V\text{,}\\ t_{j}\underset{ieV}{\sum }\left(t_{i}+wt_{i}+w_{i}+\frac{d_{ij}}{v}\right)=\forall i\in V\text{,}\\ t_{i}+wt_{i}+w_{i}+t_{ij}+\left(1-x_{ij}\right)t_{0}\leq t_{j}\forall i\in V,j\in V,i\neq j\text{,}\\ wt_{i}=\max\;\left\{0,ET_{i}-t_{i}\right\}\forall j\in V\text{.} \end{array}\text{.} \end{array}$$

Among them,

  (1) indicates the minimum total cost
  (2) indicates delivery of goods within the time window
  (3)represents the time-varying relationship of unmanned delivery cars delivering at adjacent customer points
  (4) represents the time change between points
  (5) represents the relationship between waiting time and *ET* sum

In the above equations, the unmanned car distribution near the distribution point preferentially selects the nearest distribution point according to the path. After completing the delivery, it moves on to the next nearby delivery point for delivery. The time required includes the time to the nearest delivery point, the waiting time at the delivery point, the time to the adjacent delivery point, the waiting time at the point, the time consumption due to other factors, and the final return time.

In the AVRPTW problem, the lack of energy in the middle of the unmanned delivery car is not considered for the time being. Therefore, the cost of using unmanned delivery cars for delivery mainly includes fixed costs, transportation costs, and penalty costs for violating the time window. With the gradual improvement of the delivery service, customers can make an appointment for the delivery time. This leads to the car routing problem considering time windows from the traditional car routing problem. According to the strictness of time window constraints, time windows can be divided into hard time windows, soft time windows, and mixed time windows.

The above equation is a model for car path planning and construction, which includes three path problems considering time windows. They are introduced as follows:

1. The first is the hard time window. A hard time window requires that the car must arrive within the time period predetermined by the customer. Otherwise, the customer will refuse to accept the goods. The customer satisfaction is zero, and the penalty cost is infinite.
2. The second is the soft time window. The soft time window requires the car to be delivered within the time limit scheduled by the customer as much as possible. If the scheduled time is exceeded, the customer can still accept the goods, but the delivery party needs to pay a certain penalty fee.
3. The third is the mixed time window. Mixed time windows contain the characteristics of both hard time windows and soft time windows. They require delivery within the period predetermined by the customer as much as possible, and the item must be delivered within the time period acceptable by the customer. The item is delivered within the predetermined time period, the customer satisfaction is one, and the penalty cost is zero. When the time exceeds the predetermined time limit but within an acceptable time limit, the customer satisfaction decreases as the distance between the actual arrival time and the predetermined time limit increases, and the penalty cost increases relatively. It exceeds the acceptable time limit, the customer rejects the shipment, the satisfaction is zero, and the penalty cost is infinite.

From the above, the express delivery path planning of unmanned delivery cars is a distribution issue that companies must consider when promoting unmanned delivery cars. The VRPTW model is briefly introduced based on the collected research studies on unmanned delivery cars and car path planning.

## 3. Description of the Problem of Unmanned Delivery Cars under COVID-19

**Figure 3:**
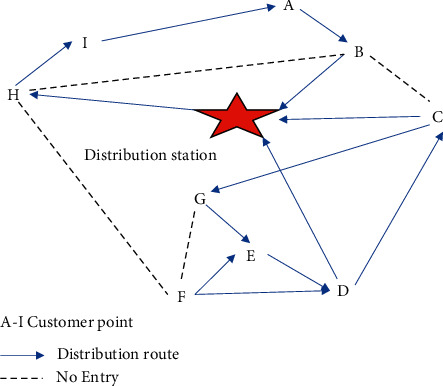
Schematic diagram of the routing problem of unmanned delivery cars.

In general, the problem of route planning for unmanned delivery cars refers to the inconvenience of personnel or the inability to complete the delivery in a specific area or at night. However, people have to isolate themselves at home after the outbreak of COVID-19, reducing the number of times they go out. Distribution becomes difficult [29]. Unmanned delivery cars are used to meet the delivery needs of customers. Under certain constraints, the delivery cost can be reduced by rationally planning and arranging cars [30]. The AVRP has the following characteristics compared with the traditional VRP. The first is that it faces the constraint of receiving time, which is the VRP with the time window. The unmanned delivery car is completely autonomously controlled by the program, so there is a fixed maximum waiting time. The second is that some sections of the road are closed to traffic for safety reasons. It is difficult for outsiders to deliver. Therefore, the distribution center uses unmanned distribution cars for distribution. However, no-passage rules have been imposed on driverless cars in some sections of the area for safety reasons. In the actual delivery process, the automatic delivery car must accept the assistance of the consignee, so it must be carried out within the customer's scheduled period as much as possible. The goods are delivered within the interval [e_*i*_, *l*_*i*_]. Waiting times for automated delivery cars are fixed. There is an acceptable time window [*E*_*i*_, *L*_*i*_] to maximize the acceptance of the goods by the customer. *E*_*i*_ represents the earliest service time that the customer can accept, and *L*_*i*_ represents the latest service time that the customer can accept. Customers who are earlier than *E*_*i*_ or later than *L*_*i*_ cannot receive the goods. In the [*E*_*i*_,*l*_*i*_], el, or [*e*_*i*_,*L*_*i*_] time range, customer satisfaction will decrease as the gap between appointment times increases, resulting in penalty costs.  Figure 3 displays a schematic diagram of the problem.

In Figure 3, the letters A–I represent the following: A, distribution station; B distribution station; C, distribution station; D, distribution station; E, distribution station; F, distribution station; G, distribution station; H, distribution station; I, distribution station.

## 4. Model Optimization

**Figure 4:**
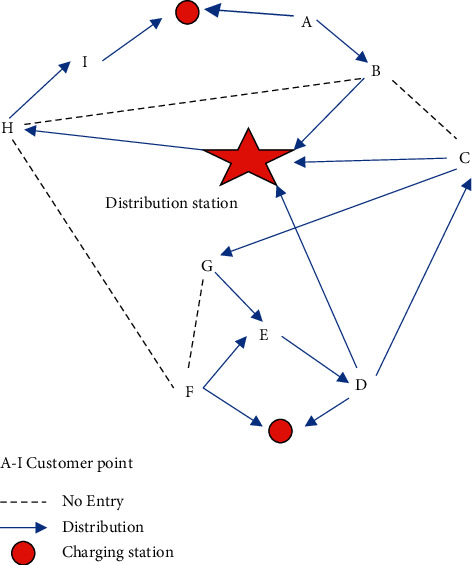
Problem diagram.

When the location of the customer point in a specific area of the city where the epidemic has occurred is determined, the delivery site must set up a unified unmanned delivery car to deliver the customer. Operating costs include fixed usage fees, transportation fees, charging fees, and fines for noncompliance. For example, this problem is shown in Figure 4.

In Figure 4, the letters A–I represent the following: A, distribution station; B, distribution station; C distribution station; D, distribution station; E, distribution station; F, distribution station; G, distribution station; H, distribution station; I, distribution station.

### 4.1. Time Window and Customer Satisfaction

**Figure 5:**
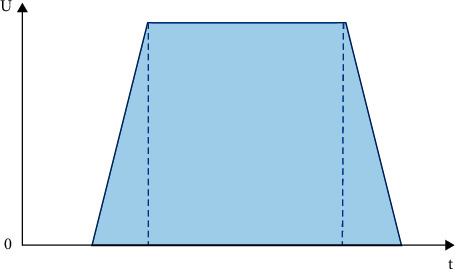
Customer satisfaction curve under the mixed time window.

It is necessary to analyze the customer's time window to minimize the total shipping costs and improve customer satisfaction. Time windows are converted to penalty fees as a measure of customer satisfaction. The mixed time window has great flexibility and meets the real-world user's demand for delivery satisfaction. Therefore, this work optimizes the route of the unmanned delivery car in the charging mode based on the mixed time window.  Figure 5 shows the customer satisfaction curve under the mixed time window.

### 4.2. Model Establishment

This problem is a multiobjective optimization problem with minimum delivery cost and maximum customer satisfaction. Therefore, the multiobjective problem must be transformed into a single-objective problem. A minimum satisfaction condition is established. Convert the maximum customer satisfaction to the maximum customer satisfaction value U, and U is not lower than the minimum customer satisfaction value, as follows:

(6) $$\begin{array}{c} U\left(t_{i}\right)\geq \delta_{i}\forall i\epsilon C\text{.} \end{array}$$

The inverse function property is used

(7) $$\begin{array}{c} e_{i}=U_{-}^{-1}\left(\delta_{i}\right)\text{,}\\ l_{i}=U_{+}^{-1}\left(\delta_{i}\right)\text{.} \end{array}$$

**Figure 6:**
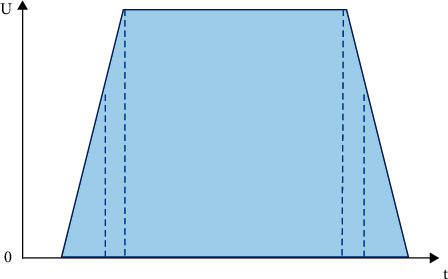
Mixed time window membership function graph based on customer satisfaction.

The constraint on customer satisfaction *U*(*t*_*i*_)≥*δ* is transformed into the time window constraint [*e*_*i*_, *l*_*i*_] on the delivery time *t*_*i*_. The corresponding relationship of *δ*_*i*_, *e*_*i*_, and *l*_*i*_ is shown in Figure 6. The time window constraint is added to the established mathematical model, which can make it a single-objective optimization model for the minimum delivery cost. Based on this, the minimum demand for customer satisfaction is gradually improved. The constraints of customer satisfaction are redefined to obtain a new distribution route planning scheme to achieve the best combination of customer satisfaction and transportation costs.

### 4.3. Solving Methods and Ideas

**Figure 7:**
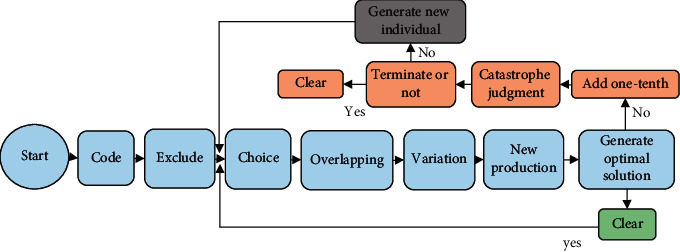
GA flowchart introducing catastrophic operation.

A new genetic algorithm (GA) is proposed for the route optimization problem of charging/commutation mode. In the construction of GA, there is a probability that it falls into the local optimum and cannot seek the global optimum. Therefore, the catastrophe operation is introduced in the construction of GA. The optimal unmanned delivery car is retained, while other individuals are randomly generated.  Figure 7 demonstrates a flowchart of the algorithm.

## 5. Experimental Steps

1. The natural number coding method is used. The customer points are converted according to the natural numbers from one to *N* and arranged and combined to form a group. Assignment sites are in units of zero and are randomly inserted across multiple populations.
2. It is determined that they meet the load capacity, cruising range, and time window constraints by calculating the load and arrival time of the group. Those who meet the conditions are accepted. Otherwise, it is rejected as an unimplementable solution.
3. Random competition is used to randomly select a pair of individuals on the roulette wheel. In both cases, the most suitable one is chosen. This selection process continues until enough people are selected.
4. The principle of self-adaptation is used, and the probability of crossover and mutation is automatically adjusted according to the adaptability of the group. In the initial stage, it is optimized to a certain extent by increasing the crossover probability and decreasing the mutation rate. Finally, the algorithm is prevented from falling into a local optimum by gradually reducing the probability of crossover and increasing the probability of mutation. *Pci*(*t*) and *Pmi*(1) represent the crossover probability and mutation probability of the *i*th individual at the *t*th iteration, respectively. *Pc*max and *Pm*max represent the set crossover probability and maximum variation probability, respectively. *f*avg represents the average fitness of the population. *f*min represents the optimal fitness of the population.
5. The research object of this work is the maximum retention method. When the probability of crossover is *Pc*, two people are selected from the entire population. A crossover point is randomly set. The gene position of this crossover point does not change, and the rest of the genes are random. When the load and time window constraints are not met, the crossover operation will be repeated until a new possible solution.
6. The reverse mutation method is used here. When the mutation probability is Pm, two individuals with the optimal solution are randomly selected, and their positions are exchanged.
7. First, the initialized Co is set, and the initial counter *T* = 0. The value of the counter *T* increases by one after each repetition. This counter will be cleared when a new best solution emerges. If the calculated *t* is greater than Co, a locally optimal solution may appear. A catastrophe operation must be performed at this time.
8. The set maximum number of iterations is determined.

## 6. Results and Discussion

### 6.1. Model Optimization Structural Analysis

The improved GA is used to solve the route optimization problem of unmanned cars in a charging state. The optimal transportation routes and car scheduling results are obtained.  Table 3 shows the optimal car scheduling cost in the charging mode, and Table 4 suggests the optimal car scheduling results in the charging mode.

The conclusion is as follows: the total power is 16.6 kWh during the allocation process of car ①. One charge is performed during the distribution of car ②, and the total power is 18.78 kWh. The remaining cars do not need to be charged during transport. The total transportation cost is 714.81 yuan, and the total running distance is 280.44 kilometers. Among them, the fixed fee for unmanned delivery cars is 120 yuan. The running cost is 28.05 yuan. The charging fee is 54.6 yuan. The penalty/opportunity fee is 512.07 yuan.

### 6.2. Effectiveness Analysis

**Figure 8:**
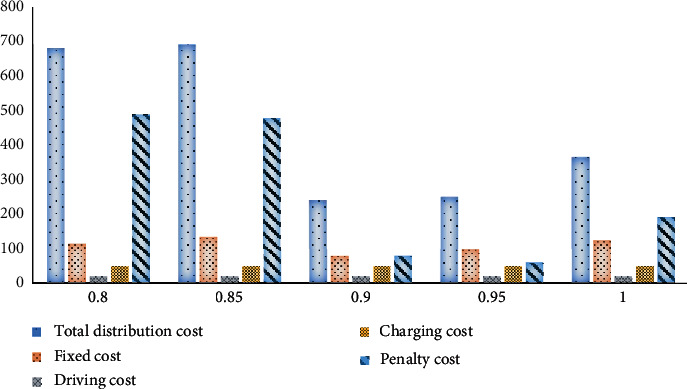
Changes in objective function values under different customer satisfaction.

The degree of customer satisfaction varies with the degree of restriction on the arrival time of unmanned delivery cars, and the charging time has a great impact. Therefore, the influence of customer satisfaction on optimization will be explored here. The value and cost of the objective function are analyzed under the conditions of 80%, 85%, 90%, 95%, and 100% customer satisfaction. The change in the objective function is shown in Figure 8.

**Figure 9:**
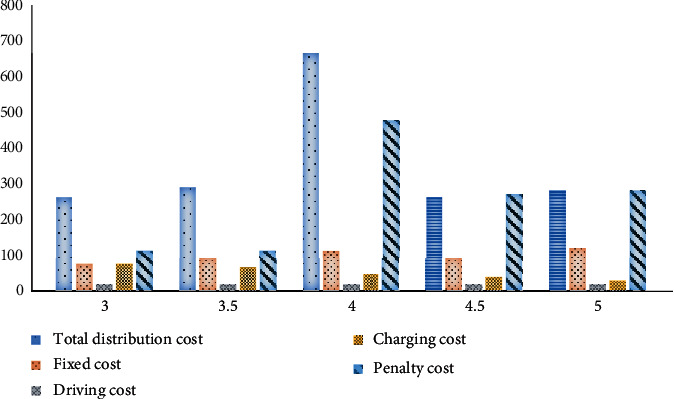
Changes in objective function values under different kWh mileage coefficients.

The mileage coefficient per kilowatt-hour refers to the distance that the car's battery can support the car's driving distance. It can reflect the energy consumption of a car to some extent. The size of this energy greatly impacts the battery life of an electric car. The battery technology of unmanned delivery cars continues to evolve, and their range will also vary according to actual needs. Here, the change of objective function value and various costs is explored under the condition of 80% customer satisfaction, the variation range of mileage per kilowatt-hour is [3, 5], and the mileage per kilowatt-hour is 3, 3.5, 4, 4.5, and 5, respectively, as shown in Figure 9.

In the actual application process in *X* city, the specific location of COVID-19 is found on the epidemic map. Traffic controls are in place at these locations, and only unmanned delivery cars can deliver supplies. Here, the change of the maximum flight time *L* of the drone is studied. Under different *β* and *L* conditions, ten procedures are performed, respectively, and the best average value is obtained.

**Figure 10:**
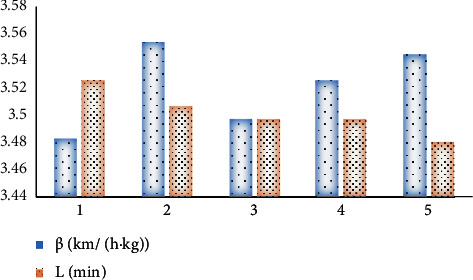
Variation of the optimal value for different *β* and *L*.

In the optimization of the transportation route, *β* or *L* will influence the optimization of the transportation route. In the process of transportation, the volume of freight greatly impacts the speed of the unmanned delivery car. The optimal solution has few objective function values. Therefore, the unmanned delivery car with little load impact and long battery life should be selected as far as possible in the actual distribution process, thereby greatly shortening the distribution time. However, this will increase the cost of unmanned delivery cars. Unmanned delivery cars can carry out “contactless” delivery compared with traditional truck delivery. The existence of external factors such as high-rise buildings will impact the safety of unmanned delivery cars. In the future, it is possible to analyze the cost of different models of unmanned delivery cars and combine them with unmanned cars to meet the needs of different situations. In addition, various operators in the adjacent search algorithm can be further studied, and its performance can be improved by designing different adjacent search operators.  Figure 10 indicates the variation of the optimal value for different *β* and *L*.

## 7. Conclusion

Unmanned delivery cars have played a big role in this epidemic. In the days to come, driverless technology will gradually become popular. However, there are still many costs and relevant laws and regulations that are not perfect, which need to be discussed deeply. A basic unmanned delivery car model based on AVRPTW is established, and the research is carried out through example verification. An optimization model of unmanned delivery car route based on the customer time window is proposed for problems such as cruising range limitation and the long charging cycle of unmanned delivery cars. Besides, an improved GA is used to solve it. The shortcomings of this work are as follows: (1) only the independent distribution of unmanned delivery cars is considered, and the collaborative delivery problem of “human + unmanned cars” can be further discussed in the future. (2) When the model is built, it is assumed that the power consumption and charging efficiency of the battery are constant. Therefore, future research should be close to reality. (3) The research done here is aimed at a special new coronavirus, and its application scope is relatively narrow.

## Figures and Tables

**Table 1:** Landing of some unmanned delivery car products in China.

Enterprise name	Unmanned car-type products	Delivery item	Reachable destination	Whether it is used during the epidemic
JD	Robot	Medicines and supplies	Logistics station and hospital	Yes
Baidu	Apollo minibus	Food	Business district	Yes
Neolithic Co., Ltd.	Unmanned car	Food	Hotels and hospitals	Yes
Meituan	Unmanned car	Food	Business and community	Yes
Yiqing	Kuafu unmanned car	Food	Logistics ports and rural areas	Yes
Xingshen Intelligent	Unmanned delivery car	Supplies	Government and directly affiliated units	Yes

Data source: it is organized according to network information.

**Table 2:** Parameters of several unmanned delivery cars.

Product name	Speed per hour	Maximum load (kg)	Farthest distance (km)	Whether the battery can be replaced	Whether it can be charged
Little donkey	Less than 20 km	100	102	Yes	No
Jedi 3000H	More than 40 km	500	100	Yes	No
Yiqing Kuafu	More than 30 km	1000	100	Yes	Yes
Real little yellow horse	3–10 km	40	90	No	Yes
Yiqing Kuafu mini	Less than 20 km	250	40	Yes	Yes

Data source: it is organized according to network information.

**Table 3:** Optimal car scheduling cost in the charging mode (unit: yuan).

Number	Driving cost	Charging cost	Penalty cost	Fixed cost
①	8.3	26.56	209.53	20
②	12.07	28.16	109.52	20
③	2.33	0	77.47	20
④	3.96	0	74.96	20
⑤	1.28	0	35.83	20
⑥	0.23	0	4.7	20
Total	28.05	54.6	512.07	120

**Table 4:** Optimal car scheduling results in the charging mode.

Number	Maximum load (kg)	Charge capacity (kWh)	Maximum delivery distance (km)
①	75.77	16.6	81.96
②	70.55	18.78	120.63
③	21.53	0	23.23
④	74.6	0	39.48
⑤	12.36	0	12.92
⑥	7.7	0	2.25
Total	262.72	35.28	280.44

*Note. δi* = 0.8.

## Data Availability

All data are fully available without restriction.
